# Co-designing a wiki-based community knowledge management system for personal science

**DOI:** 10.1098/rsos.240275

**Published:** 2024-07-10

**Authors:** Katharina Kloppenborg, Mad Price Ball, Steven Jonas, Gary Isaac Wolf, Bastian Greshake Tzovaras

**Affiliations:** ^1^ Inserm U1284, Université Paris Cité, Paris, France; ^2^ Open Humans Foundation, Sanford, NC, USA; ^3^ Quantified Self Labs, Berkeley, CA, USA; ^4^ The Alan Turing Institute, London, UK

**Keywords:** personal science, patient-led research, peer production, wikis, co-creation, citizen science

## Abstract

Personal science is the practice of addressing personally relevant health questions through self-research. Implementing personal science can be challenging, owing to the need to develop and adopt research protocols, tools and methods. While online communities can provide valuable peer support, tools for systematically accessing community knowledge are lacking. The objective of this study is to apply a participatory design process involving a community of personal science practitioners to develop a peer-produced knowledge base that supports the needs of practitioners as consumers and contributors of knowledge. The process led to the development of the Personal Science Wiki, an open repository for documenting and accessing individual self-tracking projects while facilitating the establishment of consensus knowledge. After initial design iterations and a field testing phase, we performed a user study with 21 participants to test and improve the platform, and to explore suitable information architectures. The study deepened our understanding of barriers to scaling the personal science community, established an infrastructure for knowledge management actively used by the community and provided lessons on challenges, information needs, representations and architectures to support individuals with their personal health inquiries.

## 1. Introduction

Patient-led research has gained recognition for its innovative potential in addressing unexplored research areas identified by patients [1–4]. A specific form of participant-led research is personal science, in which individuals employ empirical methods to explore health questions relevant to them [5,6]. In these self-directed *n*-of-1 studies [7], practitioners use commercially available or self-made tracking devices, design protocols and choose analysis tools to interpret their health data. As individuals perform all or most of these research steps outside traditional academic research settings, personal science can be interpreted as a form of citizen science [8–10]. Personal science can yield unique insights for individuals [11,12] and enhance their sense of agency and quality of life [13]. Moreover, self-research can drive the development of new approaches or tools [14,15], inspiring clinical research with these tools and ideas [16].

While there is no epistemic necessity to collaborate, communities nevertheless play a crucial role in personal science. Practitioners often share experiences and feedback through various channels such as online and offline meetings, forums, blogs, chats or social media [17]. Two notable communities dedicated to personal science are Quantified Self, which has pioneered the concept of personal science [5], and Open Humans. Since 2008, Quantified Self has hosted numerous online and offline ‘Show and Tell’ events in which self-researchers present their projects under the slogan ‘self-knowledge through numbers’ [18]. They maintain an active online forum and an archive of meeting recordings [5]. Quantified Self has received extensive media coverage [19], and it has been a subject of research involving health care, personal informatics [20] and its role as a cultural phenomenon [17]. The Open Humans Foundation, established in 2015 as an extension of the Harvard Personal Genomes Project [21], aims to ‘empower individuals and communities around their personal health data for education, health, and research purposes’. They manage the Open Humans platform for personal data exploration, participant-led research and citizen science [22], along with an active Slack community and weekly online community meetings, known as ‘self-research chats’.

Personal science can be intricate, as practitioners typically lead all stages of their research projects themselves and frequently need to tailor protocols, data collection and analysis tools to their specific needs and contexts [5]. Peer support serves a vital role in providing assistance. Additionally, being part of a community of peers with shared goals and values, exchanging experiences and learning from one another are key motivators for sustained engagement in self-research practice [23]. Despite active communities, as well as the acknowledged importance of peer support, there is an observed tendency for individuals to create their methods from scratch when they start a new project. This imposes barriers to the scaling of the practice to a wider audience and to learning from former projects and thus the continuous advancement of personal science knowledge. As a possible reason, lacking systematic access to community knowledge has been evoked [5]. To date, in spite of existing pathways for documentation and exchange—such as community forums, blogs and social media groups, no accumulation of a shared community knowledge base has been observed [9,16].

### 1.1. Objective

This study primarily aims to explore if and how sociotechnical interventions can support the peer production of a shared body of knowledge for personal science practitioners through a co-design process for identifying and addressing knowledge management challenges. As part of this, we additionally try to evaluate the outcomes of our design approach through different user testing methods.

## 2. Methods

In this study, we employed a mixed-methods approach to co-design and implement an infrastructure for a shared knowledge management resource for personal science. First, we engaged in a participatory design approach with an existing community of practice to identify barriers related to knowledge sharing and community support in personal science. Based on these insights, we iteratively designed and discussed potential solutions, including the development, testing and improvement of a functional prototype. Following the design phase, we conducted a user study to assess and improve the usability of the prototype, as well as to gain insights into user needs regarding the information architecture for knowledge related to personal science, using a card sorting method.

### 2.1. Design process

**Figure 1.**
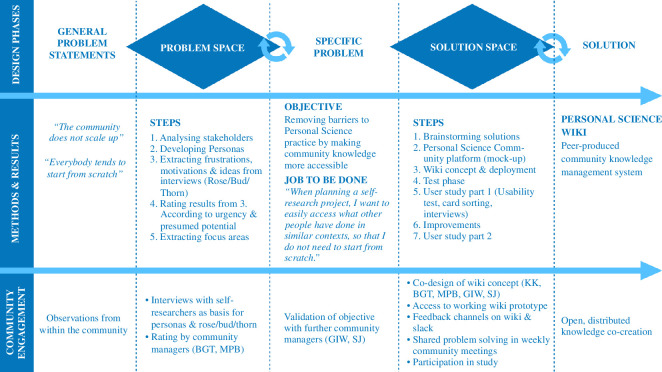
The double diamond design process (top row), alongside our used methods and results for each step (centre row) and the community engagement we performed (bottom row).

This part of the study makes use of the design thinking methodology, which serves to support matching users’ needs with technical feasibility and strategic goals [24]. More specifically, we used the ‘double diamond’ design process model [25]. This model aims to lead from a general problem statement to a specific solution, through two phases, or ‘diamonds’ (refer to figure 1). The first phase deals with exploring the ‘problem space’, in the sense of identifying a list of problems through user research, and then deciding on a specific problem to solve. The second phase is about the ‘solution space’, prototyping and user testing a range of potential solutions, until converging on one solution that will be further developed.

#### 2.1.1. Identifying stakeholders and creating user personas

Addressing the problem space first involved identifying stakeholders and considering who could be involved in the design process. To identify real community members—who could potentially provide valuable input or actively participate in the design process—we (K.K., M.P.B. and B.G.T.) brainstormed a list of active community members known to us, across levels of prior involvement. In addition to two further long-term personal science community managers (G.I.W. and S.J.)—who subsequently became more involved in this study—this also included additional stakeholders who had published academic articles about personal science, actively participated in community events or engaged through the Quantified Self forums or Slack channels. These stakeholders were identified for involvement in the design process through brainstorming or feedback sessions, and testing and engaging with prototypes. Their input was collected through weekly community meetings, asynchronously on Slack or by leaving feedback on the finally deployed prototype of the knowledge management system. Beyond testing the prototypes, this also included decision making on design decisions such as tool usage and knowledge categorizations.

Beyond this stakeholder engagement, we also created user personas which represent archetypical user types with factors like demographics, needs, wishes and challenges that can serve as mnemonic devices, making user needs prominent from the start of the design process [26]. To create personas, we engaged in a series of design iterations: initially, several team members independently created persona drafts based on interviews with real community members [23].

From these drafts, common dimensions were extracted, and lists of personas considered relevant by different team members were compiled and refined. The research team subsequently prioritized and deprioritized user personas, making use of the long-term community engagement of the four facilitators (S.J., G.I.W., M.P.B. and B.G.T.), including the knowledge of which personas already might be more frequently represented in the personal science space and which ones are more ‘adjacent’ to the community.

#### 2.1.2. From specific problems to prototypes

The same interview excerpts used for the persona creation were visualized on a collaboration board using Miro [27], clustered according to topic and categorized using the ‘rose-bud-thorn’ method [28]. Different colours were assigned to comments by interviewees, representing their motivations, frustrations and ideas or areas of potential. The outcomes were then first discussed with B.G.T. and M.P.B., who helped identify some of the most pressing problems from their perspectives as community managers of Open Humans. Based on these results, a list of potential solution approaches was brainstormed and discussed across the whole set of authors, including S.J. and G.I.W. as additional practitioner experts. This led to the development of a platform prototype mock-up using the browser-based design tool Figma [29]. The prototype’s advantages and disadvantages were discussed among the authors (refer to §3), leading to another iteration, this time incorporating a wiki approach. The overall wiki approach was then presented to the wider community stakeholders as part of the weekly ‘self-research chat’ community meetings. These meetings bring together a diverse group of personal science practitioners with different self-research priorities, which are broadly similar to our prioritized personas. In addition to a wiki-based solution, custom-made knowledge management solutions as well as approaches such as Git repositories were discussed. Building on the feedback, the wiki-based approach was selected for further development: wikis are an instance of peer production, a form of distributed knowledge production relying on self-organizing communities of individuals, notably known from Wikipedia and open-source software development [30]. This approach has been emphasized as beneficial for open-ended creative tasks and for attracting a diverse group of intrinsically motivated individuals with different skills and motivations [31]. Furthermore, wikis are widely used and appear relatively accessible to newcomers, thanks to familiarity with Wikipedia. As such, it minimizes training needs. And lastly, there are a wide range of wiki software solutions and plugins available, thus minimizing development efforts. We thus deployed a ‘Personal Science Wiki’ and made it accessible for the community to test and use.

### 2.2. User study

To assess the usability of the Personal Science Wiki, learn about user needs and to make use of the newly accumulated content to learn whether the information structures of the wiki are in line with mental models of practitioners, we conducted a mixed-methods user study. The study included a usability test, a card sorting task and a short interview, all conducted online with one participant at a time using video conferencing software. Ethical approval for this user testing was obtained from the Institutional Review Board of the French National Institute of Health and Medical Research (Inserm, IRB00003888) on 10 January 2023. Calls for participation were disseminated through the Quantified Self forum, the Open Humans Slack channel, our team’s institute’s channels, direct email invitations to community members and snowball sampling.

#### 2.2.1. Usability test

To evaluate the usability [32] of the wiki, we developed a test protocol encompassing common user tasks. As there is not a single specific workflow for effectively using the Personal Science Wiki, we designed a list of common use cases for the usability tests. This list was created by noting issues that appear to be common, based on being topics that are frequently asked/discussed during the weekly community meetings and furthermore align with the envisioned requirements of our user personas. The study protocol incorporated four information-seeking tasks from this list, covering typical self-tracking topics—as similarly based on frequent topics of discussions in community meetings—across the wiki’s main categories and using semantic linking functionality. The semantic linking allows for querying and aggregating wiki pages based on the presence of different metadata properties, such as ‘find all pages which give “sleep tracking” as their topic’. In addition, general feedback questions and two open search tasks, where participants freely explored the wiki to gather information for self-research projects, were included. The primary tasks are outlined in table 1. For each of those tasks, we considered it to be successfully achieved if the participant managed to find a corresponding page that was semantically tagged with the correct category (e.g. a ‘tool’ page that linked to the ‘sleep tracking’ topic for task number one). Participants were not given a time limit for the task; tasks were considered not successfully accomplished when participants gave up or requested help from the moderator.

**Table 1.** Tasks of the usability tests.

item no.	task/question
1	‘Please look for a device that records sleep data.’
2	‘Please look for projects other people have done related to sleep tracking.’
3	‘Please look for people who have worked on activity tracking.’
4	‘Please look for projects that use a Fitbit device.’
5	‘Imagine you would like to improve your sleep, and stumbled across this wiki. Please explore the wiki in order to see if and how it could help you to implement your project.’
6	‘Based on experience/interests of participant: imagine you would like to do a project about condition/topic X and you stumbled across this wiki. How would you try to find information that might be useful for you?’

During the study sessions, K.K. acted as the moderator to guide participants through the tasks, during which participants were instructed to open the Personal Science Wiki homepage and share their screen. We used a ‘Thinking Aloud’ protocol for the tasks, in which the moderator guided them through the search and exploration tasks sequentially, prompting them to verbalize their thoughts throughout the process. The datasets generated from these usability tests consist of transcripts from video recordings of the sessions. These transcripts were initially auto-transcribed using the tactiq browser extension [33], manually corrected and then anonymized. For each transcript, task-specific summaries were created, detailing information on task completion, ease of completion, difficulties faced, observations made and comments provided. Using these summaries, we compiled a list of usability issues and assigned priorities based on (i) how commonly participants encountered an issue; (ii) the authors’ perception of how likely it seemed that a solution would improve on a given challenge; and (iii) how easy to address any problems were. Based on this, we made corresponding adaptations to the wiki. To assess the impact of these changes, the same usability protocol and analysis were repeated with new participants, and the results from both test iterations were compared.

#### 2.2.2. Card sorting

As part of the study, a card sorting task was conducted to explore if our information architecture aligns with participants’ mental models of how they internally organize information related to personal science. Originating from psychology [34], the term ‘mental models’ refers to ‘personal, internal representations of external reality that people use to interact with the world around them’. Also framed as ‘naive theory’, they are understood as sets of causal explanations and organizations of complex systems people use in daily life [35]. They are not necessarily accurate, are unique to each person and can evolve with new experiences or learnings [36]. Mental models play a role in the development of knowledge management systems, since the structure of the system should resemble users’ mental models, so they can find information and thus efficiently use the system. Finding a suitable structure is challenging, because mental models might differ between users, and because of the evolving nature of knowledge within the system [37]. A mismatch between users’ mental models and the system architecture can cause usability issues [38].

Card sorting provides a common user research method to elicit mental models and to test or generate information architectures for online resources [39]. We used the *open* card sorting method, in which participants were given virtual ‘cards’ containing the title of content pages along with the lead text. Participants were then asked to group these cards into clusters that make sense to them and to find an overarching label for each cluster. The results help to understand the relationships and organizational structures users apply to the given content [36].

To prepare the task, we selected a subset of page titles from the Personal Science Wiki as cards. We aimed to include cards representative of the content, covering both our main content categories, as well as cards that were challenging to sort within the existing information architecture, for example, because they were viewed as fitting into more than one category or into none. Cards were identified as challenging for the current categories based on community feedback in the weekly community meetings as well as discussion among the authors. The initial list of 60 cards was reduced to 45 after a test run with a volunteer in order to prevent participant fatigue, with the card sorting task lasting 20–30 minutes depending on the participant. A selection of exemplary cards is shown in table 2. For the full list, refer to electronic supplementary material.

**Table 2.** A selection of cards used for the card sorting exercise.

card	label
a decade of tracking headaches	a decade of tracking headaches is a show and tell talk by Stephen Maher [...].the talk was given on 22/09/2018 and is about pills intake, sleep and stress.
activity tracking	activity tracking typically describes the act of tracking physical activity [1] and is frequently measured through metrics such as steps, calories burned, distance walked/run, heart rate and [...]
autoethnography using one button tracker and Jupyter notebook	this page provides a step-by-step guide on how one can use a one-button tracker such as the Puck.js to do an autoethnography that combines qualitative and quantitative data.
bangle.js	the Bangle.js [1] is the name of a series of open-source smartwatches that are made by Espruino under the leadership by Gordon Williams, who also designed the Puck.js open-source hardware that can [...]
blood glucose tracking	blood glucose tracking involves methods and tools to measure blood glucose levels, commonly in the context of diabetes but also by non-diabetic users.

Data were collected using the browser-based tool kardSort [40]. Similar to the usability test, a moderator was present through video conferencing and participants shared their screens while voicing their thoughts out loud.

Both quantitative and qualitative methods were employed for data analysis. Quantitative approaches combine and analyse data from all participants to find an ‘average’ organization of items [41]. On the other hand, qualitative approaches provide insights into the reasoning and motivation behind participants’ categorizations, and account for individual variations [42]. For the quantitative analysis, we downloaded tabular outputs from kardSort, and conducted hierarchical cluster analysis with average linkage, a commonly used approach that creates balanced, easily interpretable clusters [39,43]. For each participant, we calculate the pairwise distance between all cards, assigning a distance of zero if the participant assigned them to the same cluster or a one if they are in different clusters. These distances are then summed up across all participants and subsequently iteratively grouped into clusters from the bottom up, until all cards form one supercluster. As a result, if all participants sorted two cards together, these cards are assigned a distance of zero, and inversely, if all participants sorted two given cards in separate categories, they are assigned the maximum possible distance. In other words, cards which have a low distance do frequently appear together in the participant-generated groupings, and are thus probably belonging to a similar concept. Our code for this analysis has been packaged into the open-source Python package ‘cardsort’ [44]. For the qualitative analysis, similar to the usability tests, transcripts from video recordings were used. Excerpts from the cleaned and anonymized transcripts were manually tagged using the software taguette [45]. We created tags for each card, categories of feedback, and user-generated cluster labels.

## 3. Results

### 3.1. Design process

#### 3.1.1. User personas

At the end of our iterative design of user personas, we ended up with a final list of 13 different personas, of which our team consisting of researchers and personal science practitioners prioritized 9, deprioritizing 4 as less relevant to the objectives of this study (refer to table 3). Broadly, prioritized personas represent individuals that are willing to engage in the complex process of self-research and who are driven by an intrinsic motivation and enjoyment of working with data and technology, including experimentation and trial-and-error, and a desire to enhance their health or well-being. Personas who were not prioritized included those mainly interested in ready-to-use tools for casual tracking or data donations.

**Table 3.** An overview of all personas with descriptions.

	name	description
prioritized	Taylor the Techie	Taylor is a software engineer who is interested in playing with technology at large, including playing around with hardware. Taylor loves collecting data, even if they are not 100% sure what it could be used for.
	Dannie the Data-Nerd	Dannie is a data scientist who is always interested in learning the latest methods to analyse and visualize data. They enjoy using their own data for this, as it has the side-effect of learning about themselves. Recently, Dannie often thought about using their notebooks with other people’s data in order to compare patterns.
	Avery the Improver	Avery wants to do better and be better to live a better and healthier life. Self-tracking and self-research seem to offer useful insights for this, and Avery is keen to find out more and experiment.
	Eden the ePatient	Eden is a diabetes patient and is frustrated by the lack of research in areas of their condition. They would like to address a specific question and back it up by data of many other patients to be able to make a grounded claim in order to advocate for their case and to improve the lives of diabetic people.
	Riley the Researcher	Riley is a professional researcher and is familiar with research methodologies—using controls, alternative hypotheses. Riley is curious about and interested in applying the research methods to understanding their own life.
	Cameron the Curious	Cameron is curious about themselves and wonders how to learn more through tracking or self-research—and enjoys coming up with interesting new questions to ask and answer.
	Dylan the Discovering Patient	Dylan was recommended by their doctor to try self-monitoring in order to learn which factors influence the symptoms of their chronic migraine. This was their first point of contact with tracking and data, let alone coding! With growing interest, Dylan is starting to discover the possibilities of self-research by reading about and trying out some trackers and apps.
	Sawyer the Student	Sawyer is in their first Master year in biology and has heard about self-tracking. They are curious to what extent some of the empirical knowledge they are learning to apply to other areas could also be applied to themselves, more personally. Sawyer is also considering buying a Fitbit.
	Harper the Hobbyist	Harper worked for many years as an engineer and has always been very active in tech-related hobbies and fixing things at home. Now that they are retired and also worried about some possible hereditary health conditions, Harper wants to use part of their time to discover new things empirically.
deprioritized	Ryan the Reluctant Patient	Ryan was advised by their doctor to try self-tracking to monitor their blood pressure, nutrition and physical activity. They find it really tedious and laborious to implement a tracking routine in everyday life and want to invest as little time and effort as possible.
	Sam the Scientist	Sam is a medical researcher in the field of autoimmune diseases. They have considered using self-tracking data for some studies. They are not really interested in getting involved with the community: for their purposes, it would be best if people followed their research protocol and sent them the recorded data afterwards.
	Drew the Data Donor	Drew has been tracking their heart rate, sleep and exercise over several years and has accumulated quite some data. While not particularly interested in analysing the data for themselves beyond the conclusions they learned to draw intuitively, Drew would like to donate their data to research.
	Charlie the Casual Tracker	Charlie gradually got more into self-tracking with the advancement of the mobile devices they use in daily life. They are satisfied with the default settings and casually having a look at their step count or pulse. Charlie also finds the prompts reminding them to move more in daily life quite useful and is satisfied with their new device.

#### 3.1.2. Exploring the problem space

**Figure 2.**
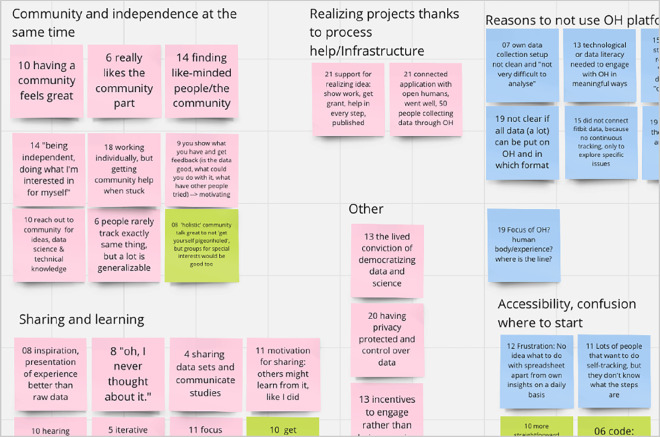
Screenshot of a part of the visual collaboration board, showing statements from interviews clustered by topic and colour-coded according to the ‘rose-bud-thorn’ method: pink—motivations; blue—frustrations; green—areas for improvement.

From the existing interviews with self-researchers, which we had also used for creating the personas [23], we extracted a sample of (shortened) excerpts related to motivations, frustrations and ideas around their self-research practices and community engagement. Put on virtual post-its, we subsequently clustered them into overarching themes, as depicted in figure 2.

Following the rose-bud-thorn method, each card was assigned a distinct colour, with pink denoting motivations, blue representing frustrations and green indicating ideas or areas of potential. For instance, a motivation was expressed as ‘Finding like-minded people/a community’, a frustration was articulated as ‘No time to attend community meetings’, and an improvement idea was presented as ‘Make code less intimidating at first sight’. We deliberated the statements and classified them into prioritized areas for the ongoing design process, areas considered long-term goals that were not immediate for this design process, and deprioritized areas. This priorization was done based on whether areas aligned with the needs of the prioritized user personas, were specific enough to personal science and fit to overall research constraints (objectives and timelines). The full list is in the electronic supplementary material. The process resulted in the identification of a main objective related to the general problem statements (‘Everybody tends to start from scratch’ and ‘The community does not scale up’), with an alternative formulation as a ‘Job-To-Be-Done’ [46], as well as a strategy focus on:

— *Objective*: Removing barriers to Personal Science practice by making community knowledge more accessible.
— *Job-To-Be-Done*: ‘When planning a self-research project, I want to easily access what other people have done in similar contexts, so that I do not need to start from scratch’.
— *Strategy*:
  (i) Focus on community as a way to form long-term social motivations.
  (ii) A kind of ‘cross-referenced informations system’ as ‘a way to find what has been done’ and as ‘something that encourages people to share their progress’.

#### 3.1.3. Prototyping

To start the ideation phase, a brainstorming session with all authors (including personal science community managers) was conducted to complement the identified focus areas with potential approaches and anticipated challenges, focusing on whether solutions were assumed to be useful to the prioritized user personas. For instance, the focus area ‘A way to find what has been done’ was enriched with ideas for a cross-referenced information system, including features such as ‘For a given topic, what methods can you use? What worked versus what did not?’ and ‘Make processes/tacit knowledge explicit’. Anticipated issues were also outlined, such as ‘Would a given approach add any benefits over existing tools/platforms that are already used by personal science practitioners (e.g. forums or Reddit)?’ and ‘How does one maintain lists of tools against spam, e.g. advertisements for apps from startups?’. Subsequently, a list of potential solution approaches was compiled, encompassing a custom personal science social platform, a community wiki, reusable programming notebooks or data management tools and a reassessment of the Open Humans platform’s usability to align user flows with personas.

The concept of a custom platform was further developed into a mock-up using Figma (refer to electronic supplementary material, figure S1). The decision was made because we assessed that the platform addresses the main objective and Job-To-Be-Done, while also emphasizing the strategy of community building and sharing. This envisaged platform was conceptualized as a social network where users could document and discover projects, seek advice and connect with individuals sharing similar interests. During a team discussion, we evaluated whether such a solution would fit the various goals and motivations that we had prioritized through the user personas and exploration of the problem space, such as sharing acquired knowledge, seeking assistance when stuck or in need of ideas for new projects, discovering what others have done and connecting with and expanding the niche community. A key concern raised was the substantial development time and resources required upfront, with no guarantee of success and community adoption. Additionally, the concept heavily relied on broad user adoption and input to become valuable, particularly in motivating self-researchers to actively contribute.

In response to these concerns, the team settled on the wiki format to enhance feasibility with minor trade-offs: given the availability of open source Wiki software implementations, this would allow cutting down resource needs at the cost of some flexibility compared with tailor-made solutions. Additionally, a wiki allows for aggregating knowledge by cross-linking and summarizing resources, without having to rely on the original knowledge creators to necessarily use the platform, which was deemed more aligned with expectations for initial user engagement. Following a discussion with the broader personal science community in a community meeting, the original idea of a custom platform was not discarded, but the wiki format was seen as a more achievable and practical approach to address the challenge of creating a cross-referenced information system.

### 3.2. Personal Science Wiki

**Figure 3.**
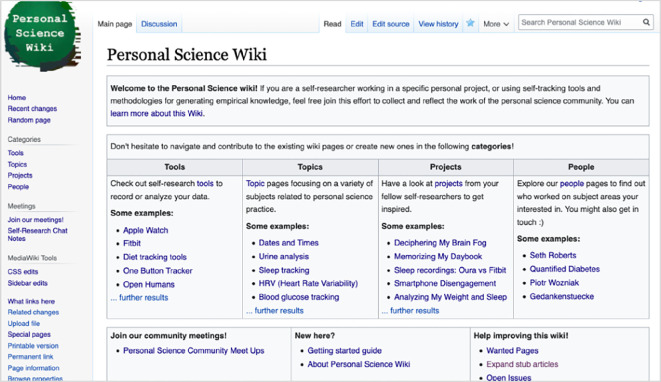
An early version of the Personal Science Wiki homepage, showing the main category structure ‘Tools’, ‘Topics’, ‘Projects’ and ‘People’.

Following this prototyping, the Personal Science Wiki was implemented as a collaborative knowledge management system for self-researchers. This wiki is accessible at https://wiki.openhumans.org. MediaWiki was chosen as the underlying technical infrastructure owing to its mature capabilities in supporting co-created knowledge management systems, its adaptability and ease of deployment, allowing us to quickly gain user feedback from real usage. An essential feature is the open editing model, potentially minimizing maintenance and moderation bottlenecks. Based on an initial brainstorming between K.K., M.P.B., and B.G.T., we structured the initial information architecture around categories that are similar to those used in the archive of Quantified Self’s Show and Tell talks, comprising Tools, Topics, Projects, and People (refer to figure 3).

**Figure 4.**
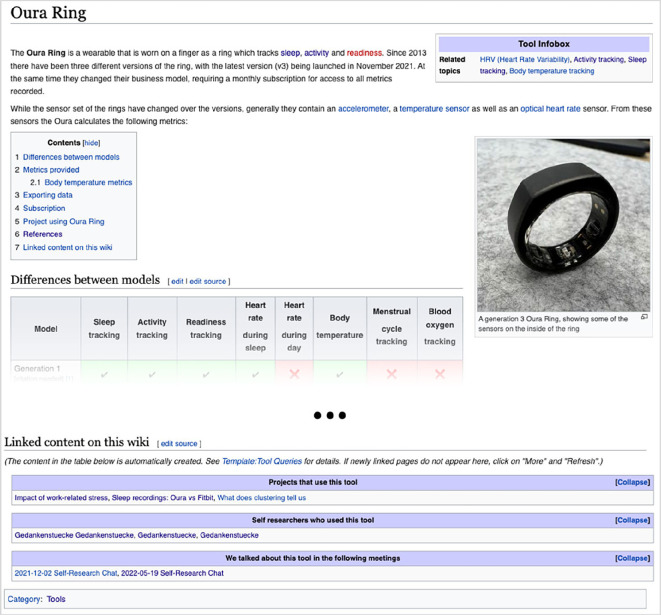
An early version of the ‘Oura Ring’ page, a page of the category ‘Tool’, including a ‘Tool Infobox’ template on the top right and a ‘Linked content on this wiki’ template on the bottom of the page.

To facilitate automatic page linking, we integrated the ‘Semantic MediaWiki’ [47] extension, an add-on for MediaWiki allowing the assignment of properties to pages. This feature enables semantic querying, allowing the aggregation of pages that share a common property. Templates were developed to use these properties, connecting content across main categories. In the ‘Infobox’ template, users can assign other pages as properties. This allows, for instance, a page describing a self-tracking tool like the Oura Ring (refer to figure 4) to be linked with associated topics such as sleep tracking or activity tracking. Additionally, we implemented a template which aggregates all pages linked back to the current page. This serves to showcase projects or self-researchers associated with a particular tool (cf. figure 4).

As a peer-produced platform reliant on user contributions, community engagement became a central focus and challenge. After developing a proof of concept, the prototype was presented to G.I.W. and S.J. to provide a sense-check. Subsequently, we invited the use of the wiki to community members who attended the weekly community meetings, encouraging the use of the early prototype of the wiki. These community members broadly covered our prioritized personas, mostly being self-research practitioners and people interested in personal science. Various pathways were established to collect user feedback: We (i) created pages on the wiki to report open issues and brainstorm ideas for improvement that any user could contribute to; (ii) provided a dedicated #wiki channel on the Open Humans Slack; and (iii) set aside parts of the weekly self-research community meetings to discuss the wiki. Of these channels, the weekly community meetings saw the most use, followed by the Wiki pages and Slack. In response, the wiki gained active usage within a small community circle. To seed the wiki with pages and include a valuable, existing source of information within our system, we imported 373 documentations of Show and Tell talks from the Quantified Self archives as project pages. After six months, by April 2022, the wiki counted 476 content pages, i.e. 100 additional user-generated content pages on top of the imported ones, and 13 users with 0 to nearly 700 edits. Notably, the most active contributor was external to our research team. In October 2022, during an online event on personal science, the wiki was officially launched to a broader audience. Between September 2021 and March 2023, the wiki featured in the notes of 56 weekly community meetings. These mentions ranged from discussing usability issues to general deliberations about the wiki approach, as well as links, when participants shared wiki pages they had edited or created.

During usage, some issues emerged. The integration of hundreds of content pages on self-research topics highlighted challenges with the existing category structure. Some pages did not align well with the main categories, leading to the ‘Topics’ category becoming a somewhat unstructured catch-all for pages that did not fit elsewhere. Additionally, formal tests of intended user flows had not been conducted, and feedback had primarily come from a small group of core members and active wiki users. This prompted the planning of a user study to assess the wiki’s usability and explore user-friendly information architectures for better structuring.

### 3.3. User study

The study was run from 23 January to 24 March 2023, with 21 participants in total, ranging in age from 19 to 66 (mean: 35 years, s.d.: 11 years). All participants were prompted to do the card sorting activity, while 10 of the participants were also asked to engage with the usability study in the same session, following the card sorting. Five of these user tests were done in an initial set of usabildr5ity tests, and another five in a second iteration after changes had been made based on the results of the first set. A description of the sample can be found in table 4.

The study also included questions about experience and interest in self-tracking, as well as wikis, including the Personal Science Wiki. The replies are summarized in table 5.

**Table 4.** Demographic information of the study sample.

demographic category	frequency	percentage
gender		
male	12	57.14
female	9	42.86
education		
PhD	6	28.57
Master	9	42.86
high school	6	28.57
professional background (several categories possible)		
engineering/research	19	90.48
computer science	9	42.86
biology	4	19.05
experience in health domain	4	19.05
geographic location		
Europe	14	66.67
North America	5	23.81
Eastern Asia	1	4.76
Southern Asia	1	4.76

**Table 5.** Study participants’ self-reported involvement in self-research.

demographic category	frequency	percentage
self-tracking experience		
none/almost none	2	9.52
casual tracking	11	52.38
intensive tracking/self-research	8	38.10
personal science community involvement (Quantified Self/Open Humans		
active involvement	7	33.34
passive/casual involvement	3	14.29
no involvement	11	52.38
involvement in other adjacent communities	3	14.29
self-tracking areas of interest (multiple answers possible)		
general fitness (physical activity, heart rate, …)	9	42.86
mental health	6	28.57
sleep	5	23.81
diet	3	14.29
productivity	3	14.29
chronic conditions	2	9.52
meta research on personal science	2	9.52
menstrual tracking	1	4.76
location	1	4.76
microbiome	1	4.76

Overall, we find that our participant population was well aligned with our prioritized user personas: motivations for participants to engage in self-tracking or self-research included monitoring, identifying patterns, pursuing specific goals, such as weight loss, an interest in trying interventions and exploring the possibilities of technology to impact one’s health, as well as meta-interests in personal science practice. To gain insights into participants’ experiences and challenges with existing information sources, other questions targeted recent experiences and satisfaction with information resources related to personal science. Participants reported seeking information for various purposes, such as deciding on which tracking devices or software to purchase or download, learning how to use them and gaining a general understanding of tracking topics and how to track specific variables. Participants turned to various sources, including online searches, scientific articles, blog articles, podcasts, Open Humans or Quantified Self forums, platforms like Reddit, advice from friends and, more recently, artificial intelligence chatbots like ChatGPT or Bing Chat for personal science-related information. While seven participants expressed overall satisfaction, criticisms included concerns about the lack of trustworthy resources due to advertising, a shortage of unbiased information or research-backed systematic analyses and conflicting opinions and reviews. Some participants also struggled to find practical information on certain topics. Notably, four participants identified a lack of information about personal science or self-research projects beyond simple monitoring. This included missing details on formulating research questions, interventions to address these questions, the connection between personal experiences and used methods or lack of information about the practice at all.

#### 3.3.1. Usability test

In the initial round of usability tests, 75% of the specific tasks were successfully completed (refer to electronic supplementary material, table S5). Successful completion was defined as a participant finding the requested piece of information within a few minutes, without giving up or requesting a hint/support by the study lead. Forty-five per cent of tasks were completed ‘fast’, meaning without hesitation or having to explore different pathways before finding the piece of information. Incomplete or slow task completion was attributed to a lack of information scent on the homepage, such as the absence of keywords like ‘sleep’, causing confusion about where to find sleep-related projects or tools. Additionally, links to the main category pages were often overlooked on the homepage, causing participants to miss opportunities to discover pages based on category tags. Subsequently, participants attempted to use search expressions like ‘projects sleep tracking’ in the search bar, leading to sometimes unspecific search results owing to the built-in mediawiki search function not being optimized for complex search terms. Another common issue was the difficulty in finding the section ‘Linked content on this wiki’ as participants did not scroll down to the bottom of the page. Participants expressed that they did not expect important content below the ‘reference’ section.

**Figure 5.**
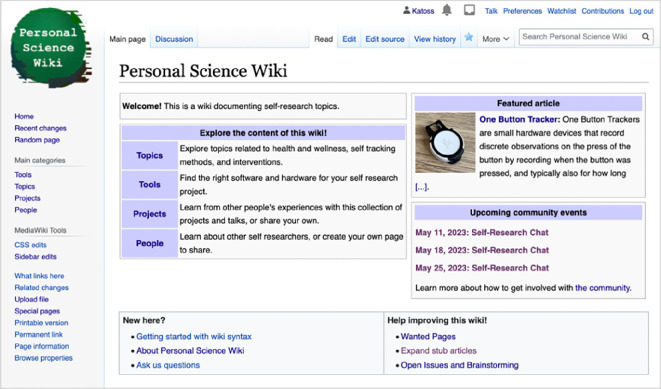
Screenshot of the updated user interface of the Personal Science Wiki homepage after the first iteration of usability tests.

In response, the interface of the homepage was rearranged to prominently display links to the main categories. The lists showing example pages were removed, as they misled some participants into thinking they represented all available content. Additionally, content showcasing community activities, an automatically updating calendar of upcoming events, and a featured article were added in response to frequent requests to find how to engage more with the community beyond the wiki. A screenshot of the updated homepage can be found in figure 5.

**Figure 6.**
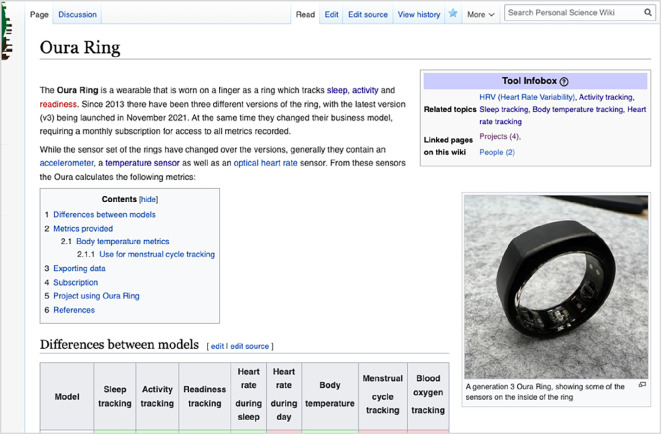
Screenshot of the updated ‘Oura Ring’ page, used as an example for a Tool page.

Moreover, we made adjustments to the templates for the semantic properties on content pages. Recognizing that most participants did not find the ‘Linked content on this wiki’ section at the bottom of the page, we relocated this section to the top right of the page within the infobox template. In this revised version, instead of displaying the entire list of connected pages, we opted to show only the count of linked pages for improved readability (refer to figure 6). Users can access the detailed list with a click.

One big challenge we identified as a priority throughout our co-design process was the findability of existing personal science knowledge. As such, a primary use that the wiki aims to facilitate is the discovery of content, such as projects or tools, related to a specific topic of interest, like sleep or activity tracking. The key entry point for this is consulting the respective topic page and exploring linked content from there. To offer an alternative pathway to discover linked content, we introduced subcategories to the main categories. The creation of subcategories was informed by feedback from usability tests and early qualitative analysis of results from card sorting tasks. We noticed during the usability test that participants expected subcategories of health-tracking topics that would lead them to content (e.g. tools related to sleep as a subcategory of tools), and that they would hesitate if they did not find them. Furthermore, participants frequently made comments during card sorting about subcategories they would like to assign. Additionally, we made use of category tags that had been assigned to Show and Tell talks in the Quantified Self archive, by clustering similar tags into overarching topics that served as subcategories in the ‘Project’ category. The new subcategories were then programmatically assigned to the pages using the MediaWiki Application Programming Interface (API). For the ‘People’ category, we opted not to add further subcategories owing to the limited number of pages in the category, suggesting that labelling project pages by individuals might suffice. Table 6 provides a comprehensive list of all new subcategories.

**Table 6.** Subcategories created on the wiki after the first iteration of usability tests.

top-level category	subcategories
topics	blood testing and tracking tools; body temperature tracking tools; data analysis tools; diet tracking tools; fitness and heart rate tracking tools; hardware; mental health, journaling and self-report tools; open source tools; productivity, learning and cognitive abilities tools; sleep tracking tools; software
tools	data analysis; disease, pain and chronic condition; discussions; experiment design; interventions; Personal Science Community; things to track
projects	body measurement projects; cognition and learning projects; diet, digestion and weight loss projects; disease, pain and chronic condition projects; environment projects; fitness and physical activity projects; habits projects; heart rate and cardiovascular health projects; how to’s; menstrual health, fertility and pregnancy projects; mental health projects; productivity projects; sleep projects; Show and Tell (*note: category existed before the usability test*); social life and social media projects
people	—

After implementing these changes, the second iteration of usability tests saw an improvement in task completion for specific search tasks, rising from 75% to 100%. Sixty-five per cent of the tasks were completed fast, without hesitation or trying several paths. Most of the hesitation in this round was owing to the task ‘Please find people who have worked on activity tracking’. Four out of five participants hesitated upon realizing that the ‘People’ category lacked subcategories, unlike the other categories. Ultimately, they navigated to the aggregation through the ‘activity tracking’ topic page, which all participants successfully located.

In the exploration tasks related to a sleep project and a topic of choice, additional insights into usability and user interests, needs and expectations emerged. For the sleep tracking project, 9 out of 10 participants began by visiting the sleep tracking topic page and exploring linked content from there. One participant initially went to the Projects main category and consulted the sleep tracking projects subcategory. Participants visiting the sleep tracking topic page expressed the desire for an overview of the topic, including information about interventions, factors impacting sleep and tracking devices. However, they lamented the lack of curated content on the page during the study.

Four participants found the list of linked sleep projects overwhelming (78 projects), struggling to determine which projects were the most relevant. The creative titles of project pages, imported from the Show and Tell talk archive (e.g. ‘Grandma was a lifelogger’, ‘Sleep as a galaxy’), were not perceived as helpful. Participants expressed a wish for a hierarchy or curated list. Regarding video recordings of project talks, participants wanted extracted information in a wiki-how style or a table containing details like methods, variables, goals and results.

Nine out of 10 participants repeated the task covering the following topics of interest: Apple Watch, blood glucose tracking/diabetes, note-taking and diaries, diet tracking, chronic pain, weight gain or loss, microbiome and cognitive testing. All topics were present on the wiki, but some lacked extensive content. Participants appreciated detailed information about devices and sensors, including projects and specific details like nutrients. Missing information about tools prompted participants to express a desire to contribute. After the study, one participant added details to the Apple Watch page, and another participant added a new page for a note-taking tool.

#### 3.3.2. Card sorting

All 21 participants engaged in the card sorting task. The datasets for analysis included transcripts of video recordings for qualitative analysis and tabular kardSort output for quantitative analysis. In line with best practices for card sorting [42], data from one participant were excluded from the quantitative data owing to creating a ‘miscellaneous’ category that can bias the results. However, their comments were still considered for qualitative analysis.

**Figure 7.**
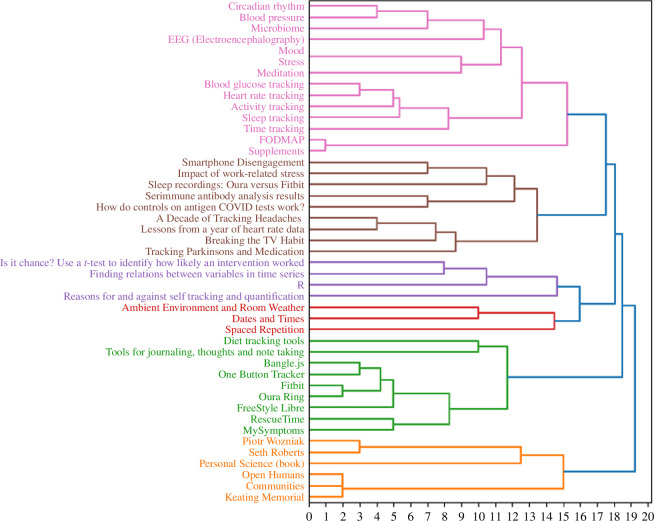
Dendrogram visualizing the results of hierarchical cluster analysis of the card sorting outputs of all 20 participants.

Regarding the quantitative analysis, 20 participants created a total of 147 categories (mean = 8.4; s.d. = 1.8; range = 5–12). The results of the hierarchical clustering are depicted in a dendrogram in figure 7. The colour cut-off was set at 80% or a distance of 16. This decision was based on perceiving clusters containing cards similar enough to represent a unique content category and distinct enough from other categories. This resulted in six categories with 3–14 cards, averaging 8 cards each.

As a next step, participant-generated labels were examined to comprehend the concepts covered in these clusters. Since hierarchical cluster analysis represents an average mental model, it is possible that no participant exactly replicated the clusters seen in the dendrogram. To still get an impression of participant-generated labels for the clusters, the following method was used: for each cluster, we first tried to see if there was a label assigned by a user for the whole set of cards. The same was repeated for subsets of cards in that cluster, removing one or more cards or subclusters. This allowed us to identify both concepts that roughly cover complete clusters, as well as concepts for subclusters in some cases. To identify concepts independent of exact wording, we clustered the labels based on similarity and tried to find overarching descriptions. A list of labels and subclusters can be found in the electronic supplementary material. Based on the results, the clusters (in chronological order) are described as follows:

— *Pink*: Labels are primarily related to tracking methods or variables to track (frequently related to the body); subclusters cover interventions or personal improvement, health topics, mental health, as well as diet and nutrition.
— *Brown*: Predominantly labelled as projects or experiments, but also as discussions or lessons learned, resources/media/Show and Tell talk or community blog; subclusters cover mental health and specific health problems.
— *Violet*: Labels largely refer to methods, with a focus on data analysis, but also research questions or tools; other concepts that appear frequently are asked questions, general skills and self-tracking in general, studies, community, advice or blog, resources and references.
— *Red*: Labels refer to parameters, variables or entities, but also to methods; the card ‘Spaced repetition’ also received labels regarding cognitive abilities or interventions.
— *Green*: Predominantly labelled as tools, including devices and apps, sometimes with keywords regarding measurement or tracking; another concept covered is tracking methods; subclusters regarding topics like general health and fitness or mental health.
— *Orange*: Predominantly labelled community, people, self-researchers; sometimes resources, references or meta-information.

The qualitative analysis of the interview transcripts provided insights into the reasonings, difficulties and organizational schemes used by individual participants. Some individual cards were occasionally criticized for covering unknown concepts (e.g. the ‘FODMAP’ diet), being too broad to anticipate the content it represents on the wiki (e.g. ‘Stress’) or being challenging to place because they were perceived as a single instance of a potentially different category (e.g. the statistical programming language ‘R’). One participant questioned if some pages should be on the wiki at all, referring to general information on topics like the FODMAP diet, which they believed was more suitable for Wikipedia than a Personal Science Wiki.

Participants frequently expressed the desire to create hierarchies of categories, which is not possible with kardSort. For example, they suggested splitting the tools category into data collection and data analysis tools, software and hardware, or tools divided by variables they track. Another suggestion was to split projects by body part or health topic and divide general health and well-being topics into subcategories regarding e.g. chronic conditions. Participants frequently mentioned the coexistence of different organizational schemes, suggesting the possibility of sorting pages either by the type of resource (e.g. tool and project) or by subject matter (e.g. fitness and chronic condition). A participant with diabetes highlighted that a dedicated ‘diabetes’ category would be straightforward and useful for them, but topics like ‘blood glucose tracking’ could also be sorted into a different health-tracking category for users with other interests. Participants also proposed different entry points to information based on users’ interests and levels of experience. For example, they suggested a dedicated pathway for newcomers to guide them through foundational knowledge before delving into specific topics, as well as a shortened path for experts to quickly find specific information. Detailed results of the user study can be found in the electronic supplementary material.

## 4. Discussion

Here, we describe how we explored sociotechnical solutions to overcome individual barriers to engaging in the practice of personal science by taking a design thinking approach to identify challenges and potential solutions. In particular, our work resulted in the use of participatory design principles to investigate how technical infrastructure for knowledge peer production could help overcome these challenges, while also trying to evaluate whether our implemented solution is generally fit for purpose through different user testing methods.

### 4.1. Identifying barriers to personal science practice

Early in our work, we defined the particular type of scaling we aimed to address in our design process, focusing on facilitating scalability to enable more individuals to participate in the entire breadth of personal science practice, from questioning to discovery. While other scaling approaches, such as automating common processes and data donation, were considered, they were ultimately discarded. Automating common processes, centred on providing ready-to-use tools to eliminate technical barriers, had been explored by the Open Humans platform, offering data import connectors, uploaders for various data sources and a Jupyter notebook integration [22]. However, this approach was dismissed owing to potential challenges in maintaining it at a larger scale, given the frequent changes in APIs or tools. Similarly, for data donation, where individuals contribute personal data to a collective dataset for scientific studies [48], Open Humans already provides tools.

Next, we identified barriers faced by individuals who want to engage in personal science, by going through a set of existing interviews with people interested in engaging in personal science, leading to the creation of user personas and further community input from personal science community managers. Going through the rose-bud-thorn method to identify priority areas, we recognized the importance of skill building. Addressing personal health questions demands a comprehensive set of research skills, including developing research questions, selecting tools, data collection, analysis, interpretation and method adaptation to individual contexts [49]. Individuals frequently rely on peer support to get directions and acquire skills, and belonging to and sharing experiences with a community was often cited as a strong motivator for continuous engagement in the practice. However, we identified some issues with the current community knowledge-sharing practices: many individuals do not have time to attend regular community meetings and these meetings are not easily scalable to accommodate a large number of participants for discussing individual projects. While asynchronous, text-based alternatives like forums, blogs, social media or chats exist, these thread-based approaches have limited capabilities for creating consensus knowledge, often remaining as a collection of individual approaches. Attempts to create overviews and best practices encounter maintenance issues and quickly become outdated, not reaching the status of a shared community knowledge base [16]. The popular narrative format of Show and Tell talks faces challenges in revealing failed attempts and iterations, as the narrative makes it seem as if the process had been streamlined from the start. Lastly, the user study highlighted people’s frustration with the lack of reliable online resources, encountering conflicting opinions and advertising when seeking self-tracking information. These findings provide insight into possible reasons why self-researchers tend to create their methods from scratch as they undertake projects.

### 4.2. Addressing the barriers with a wiki approach

After some design iterations, we opted for a wiki-based approach to provide an infrastructure for a shared community knowledge base. The Personal Science Wiki is thought to complement existing platforms, as a permanent, open, community-owned space for curating and preserving content, connecting pages in a web of semantic links. Wikis facilitate the creation of consensus knowledge by allowing multiple users to edit pages and engage in discussions through attached ‘Talk’ pages. Anyone can edit any page, alleviating maintenance bottlenecks by not depending on specific individuals. Entry barriers are low, with no mandatory user account creation [50] and the flexibility to perform minimal edits or create entire pages. Early contributors can benefit from the wiki as a space to document and share projects without depending on input from others [51]. Moreover, wiki approaches have been chosen in similar contexts such as chronic pain management [52] or DIY research projects in environmental justice [53]. Wikis offer a quick and easy set-up, facilitating the gathering of real user feedback on a functional prototype. This aligns with the recommended approach in tool design, prioritizing practical user experience over abstract discussions [54]. This also fits the development of personal research projects, particular for the user types we prioritized, who are often at the beginning of the research design phase, and the values within the personal science community, peer support, sharing, transparency and empowerment. The Personal Science community can be viewed as a community of volatile practice [55], an online community characterized by its emergent nature and continuous assimilation of knowledge from various disciplines and occupations, including academic and non-academic literature, as well as personal experiences. In fields experiencing emerging knowledge, such as during the COVID−19 pandemic, collaborative knowledge creation on Wikipedia has been found to be successful, matching the pace of scientific advancements [56]. A key difference, and an additional challenge for the Personal Science Wiki, lies in its reliance on individual experiences as a source of knowledge, which can be documented on the wiki as project pages. Consensus knowledge must then be distilled from common elements of these experiences in subsequent steps. This contrasts with Wikipedia or Wikidata, where a general notability requirement must be met for pages to be accepted initially [57]. To facilitate knowledge sharing in communities of volatile practice, Kou *et al*. [55] suggest analysing the various social roles within the community and providing dedicated features to support them. They also propose introducing a technology steward role to address the community’s technological needs, which could be a focus for future research.

### 4.3. Lessons on information needs and representation

The usability test served a dual purpose by addressing and resolving wiki infrastructure usability issues while uncovering broader user requirements for personal science information resources. During the open exploration task, participants demonstrated a realistic understanding of the wiki’s content scope, choosing topics within its existing coverage. This suggested that even at its current scale, the wiki covers common areas of interest in health and well-being tracking.

Personal science often involves tacit knowledge—skills and know-how acquired through practice [58], the transfer of which is a common knowledge management challenge [59]. During the usability tests, a number of additional challenges emerged that we had not accounted for during our co-design phase: participants frequently expressed the wish for an entry point providing an overview of each tracking topic, covering common variables or tracking tools, best practices or interventions. They also wondered about curated lists of a few exemplary projects for each topic, caused by the overwhelming nature of the existing, automatically aggregated project lists based on tags. While participants appreciated the richness of project information, they criticized the current tagging system for lacking clarity in distinguishing projects specifically focused on an aspect (e.g. sleep) or those that just track that aspect as one variable of many. The titles of Show and Tell projects were deemed too open to quickly understand what the project was about. Standardization could be a means to enhance clarity. Finally, Show and Tell, a popular talk format in the Personal Science community to share projects, faced challenges when integrated into the wiki. Show and Tell is an instance of storytelling, which has been recognized as a component of holistic knowledge management, being a mechanism for facilitating the transformation of explicit to tacit knowledge that can help to internalize other people’s experiences [60,61]. While suitable in the event contexts that these talks usually take place in, in the wiki, participants had issues to quickly identify which projects were relevant to their situation. They suggested summary tables or manuals alongside the talk transcripts for quick access to relevant information. It might point towards a bias in the community members we engaged in co-creation that these challenges only emerged during our usability testing: more experienced personal science practitioners already have some of the tacit knowledge required to navigate information in the presented representation. However, since all of these pages can be edited in a wiki, features like summary tables can be added retrospectively, creating a direction for future work. Lastly, lack of specific pieces of information regarding their topics of interest motivated some participants to add this information to the wiki themselves during or after the study. This highlights the potential of the low entry barriers of wikis to increase casual contributions and improve low-quality contributions, as outlined by Hill & Shaw [50].

### 4.4. Lessons on information architectures

The card sorting study provided valuable insights into users’ mental models regarding how they organize health-related self-research information, offering lessons for structuring knowledge resources like the Personal Science Wiki. While the initial category system (Tools, Topics, Projects, People) roughly represented mental models, participants provided input for improvement. The findings suggest that the broad ‘Topics’ category could be split into a category covering ‘health topics’ or ‘tracking topics’ as well as methods, especially regarding data analysis, interventions and community-related resources. Participants also expressed a desire to introduce hierarchies, suggesting potential subcategories like, for example, general fitness and chronic conditions for health topics, hardware and software for tools, and physical and psychological variables. Another lesson was that organizational schemes varied not only between individuals but also within individuals. Participants often considered cards to belong to multiple categories, reflecting the flexibility and complexity inherent in organizing information. Two main organizational schemes emerged: ordering pages by ‘health topic’, such as diabetes or heart rate tracking, and by ‘type of resource’, more similar to the original category structure. Quantitative analysis favoured the organization by the type of resource, with emerging subcategories for health topics. Importantly, the study suggested the use of complementary models, recognizing that a single, one-size-fits-all solution might not exist. This is in line with recommendations from the W3C consortium, suggesting the provision of multiple pathways to create robust navigation experiences [62]. Participants emphasized the importance of flexible organizational schemes, allowing users to choose based on their interests and skill levels, suggesting the need for entry points for newcomers offering overviews of general information and shortcuts for experts. A more faceted search approach could allow for such dynamic organization, but can be hard to put into practice, especially when relying on widely used standard tools such as MediaWiki. Another factor to consider is that with a growing content base, requirements to the information architecture might change. This underscores an advantage of the wiki approach, which allows users to modify and create new categories, fostering the development of ‘folksonomies’ [63]—community-created organization themes that can evolve with the growth of the resource.

### 4.5. Limitations and future work

One limitation of this study is the startup challenge faced by the Personal Science Wiki, a common issue in many peer production projects: generating content requires buy-in from a sufficient number of contributors, while having enough content is crucial for attracting and retaining those contributors [64]. However, there are promising indicators for existing use cases and the adoption of the wiki, despite the currently small user base. Over 2 years, there has been active and continued engagement from a small circle of contributors, with links to pages frequently shared in community meetings. The wiki has occasionally been recommended as a knowledge resource for personal science on other social platforms. Furthermore, issues with the Show and Tell talk format surfaced during integration and testing in the wiki, pointing towards potential future research directions. Future work should focus on extracting relevant keywords and procedural information from these talks. Additionally, field studies involving individuals planning self-research projects can provide deeper insights into information needs outside of a theoretical, lab context, as seen in the user study. Lastly, the study results can serve to refine and test new information architectures for personal science information.

Apart from that, the wiki’s co-design and testing were conducted with a specific target group—highly educated individuals predominantly residing in the global north, with a professional background in engineering and research. While this is representative of typical profiles engaging in this practice [65,66], it does not provide insights into the requirements and accessibility of the wiki for other potential user groups. Future research should consider the requirements of different demographics, enhancing accessibility for a more diverse audience interested in personal health inquiry. Lastly, at the current scale, governance, moderation and efforts to prevent misinformation were minimal. However, potential growth and the inclusion of new target groups may pose challenges that need careful consideration in the future.

## 5. Conclusion

Using a design thinking approach, we identified that peer support can offer individuals help in overcoming barriers to practising personal science, including the lack of systematic access to community knowledge. To address this, we engaged in participatory design and co-designed a wiki-based infrastructure as a shared knowledge base, aimed at alleviating maintenance bottlenecks and enabling community-created project documentation and consensus knowledge.

Through our user study, we discovered that individuals expect overviews and curated information about tracking and intervention tools, approaches and projects, with the documentation of self-tracking projects being a unique selling point of the wiki. We found that the frequently used Show and Tell talk format for project documentation poses some challenges when transferred to a knowledge management system. Issues include choosing informative titles and tags to aid in identifying relevant projects, as well as a lack of means to quickly extract information regarding research questions, methods, and outcomes.

The topics present on the wiki align with user expectations, but the depth of content is still in need of improvement through more extensive user contributions. Regarding information architectures, our results indicate the benefits of using multiple organization schemes, notably ordered by resource type and tracking or health topic area, to facilitate discovery and error recovery. Similarly, providing different entry points for users with varying levels of expertise and search use cases, as well as using category hierarchies, can enhance information scent. Future research efforts are needed to explore how these knowledge organization challenges of self-researchers’ information needs can be overcome, in particular prioritizing under-represented demographics to broaden access to personal health inquiry.

## Data Availability

Data and supplementary materials have been archived within the Zenodo repository [67]. Supplementary material is available online [68].
